# A Single-Shot Scattering Medium Imaging Method via Bispectrum Truncation

**DOI:** 10.3390/s24062002

**Published:** 2024-03-21

**Authors:** Yuting Han, Honghai Shen, Fang Yuan, Tianxiang Ma, Pengzhang Dai, Yang Sun, Hairong Chu

**Affiliations:** 1 State Key Laboratory of Dynamic Optical Imaging and Measurement, Changchun Institute of Optics, Fine Mechanics and Physics, Chinese Academy of Sciences, Changchun 130033, China; 2 University of Chinese Academy of Sciences, Beijing 100039, China

**Keywords:** speckle imaging, speckle autocorrelation, phase recovery algorithm, bispectrum truncation

## Abstract

Imaging using scattering media is a very important yet challenging technology. As one of the most widely used scattering imaging methods, speckle autocorrelation technology has important applications in several fields. However, traditional speckle autocorrelation imaging methods usually use iterative phase recovery algorithms to obtain the Fourier phase of hidden objects, posing issues such as large data calculation volumes and uncertain reconstruction results. Here, we propose a single-shot scattering imaging method based on the bispectrum truncation method. The bispectrum analysis is utilized for hidden object phase recovery, the truncation method is used to avoid the computation of redundant data when calculating the bispectrum data, and the method is experimentally verified. The experimental results show that our method does not require uncertain iterative calculations and can reduce the bispectrum data computation by more than 80% by adjusting the truncation factor without damaging the imaging quality, which greatly improves imaging efficiency. This method paves the way for rapid imaging through scattering media and brings benefits for imaging in dynamic situations.

## 1. Introduction

When light passes through mediums such as clouds, turbid water, and biomedical tissues, photons are strongly scattered as they travel through the interior of the medium, and the intensity and phase information they carry will be destroyed, making it impossible for traditional imaging methods to receive information about the object through direct observation. In recent years, high-quality optical imaging using scattering media has been found to be of great significance in the fields of biomedicine, astronomical remote sensing, and deep-sea exploration [1,2,3,4], and many computational imaging methods have been proposed.

Wavefront shaping techniques based on the idea of optical transmission matrices have been widely studied [5], such as optical phase conjugation technology using inverted input light fields for reconstruction [6,7], where the optimal wavefront is obtained through an iterative algorithm to achieve imaging wavefront shaping technology based on feedback optimization [8,9,10]; the optical transmission matrix technique that utilizes the matrix to link the incident light field and the outgoing light fields [11,12]; and so on. But the technique in most cases requires the use of digital wavefront shaping devices such as spatial light modulators (SLMs). The optical path structure is complex and requires feedback modulation and calibration to the extent that it is not applicable to dynamic observations. To solve these problems, researchers have proposed various scattering imaging techniques based on the optical memory effect (OME). Compared with wavefront shaping technology, scattering imaging technology based on optical memory effects is non-invasive and has lower requirements for light sources, media, and systems. These include speckle autocorrelation imaging (SAI) [13], imaging techniques based on the point spread function [14,15], speckle cross-correlation imaging technology [16,17,18], and scattering imaging methods based on deep learning [19,20,21]. Among them, SAI is considered one of the most promising scatter imaging techniques because of its simple operation and single-shot processing method that can obtain the object information hidden behind the scattering medium.

However, the shortcomings of this method are also obvious: the imaging field of view (FOV) is limited by the range of the optical memory effect. Many methods and techniques for extending the optical memory effect have emerged, such as techniques that utilize reference objects [17] or a priori knowledge [22] to achieve imaging in the range of the super-memory effect, and depth-of-field extensions for imaging through scattering media by means of optical field estimation [23] or speckle estimation [24]. In addition to the above techniques, imaging under strong ambient light interference conditions [25], imaging of dynamic objects hidden behind scattering media [26,27], multispectral imaging [15], 3D optical memory effect imaging [28], and imaging by combining scattering correlation techniques with compressed sensing techniques [29,30] and binocular vision techniques [31,32] have also been realized.

The traditional SAI method obtains the Fourier amplitude information of a hidden object by calculating the autocorrelation of the camera speckle and combining it with the Wiener–Khinchin theorem. The obtained Fourier amplitude information is combined with the phase recovery algorithm, the Fourier phase of the hidden object is obtained through iterative computation after setting a random initial value, and finally the reconstruction of the hidden object is realized. However, existing SAI techniques can only recover the object’s amplitude spectrum by recording the speckle intensity distribution, and the object’s phase information needs to be reconstructed by a phase recovery algorithm. Phase retrieval algorithms with iterative computation are mostly used nowadays, such as the Fienup-type phase recovery algorithm and the PR-SAMP phase recovery algorithm. This type of algorithm sets a random matrix as the initial input of the phase distribution and then seeks the best phase information that can achieve convergence through repeated iterations. But the disadvantage is that the randomness of the initial iterative phase leads to the randomness of the final reconstruction object in direction and spatial position, so that the final reconstruction result is not deterministic. To overcome these problems, inspired by astronomical techniques, Wu et al. proposed an approach to the bispectrum analysis of a single-shot speckle pattern that would obtain a defined object’s Fourier phase [33]. This method avoids a large number of computation iterations and uncertainty in the recovery results. However, the bispectrum computation of a 2D image is followed by 4D data, which are usually very large, so it is necessary to consider how to minimize the amount of bispectrum data without affecting the phase recovery.

In this study, we present a bispectrum imaging framework based on the truncation method. This method recovers the amplitude spectrum of an object from the autocorrelation of a speckle image. The Fourier phase of a hidden object is obtained through bispectrum analysis of the scattering pattern, and bispectrum truncation is used to avoid the computation of redundant data when computing bispectrum data for phase reconstruction. Finally, the phase and the power spectrum are combined and Fourier-inverse-transformed to reconstruct the object image. Compared with existing research, the method demonstrated in this study can greatly reduce the amount of computation and quickly recover hidden objects without damaging the recovery quality.

## 2. Method

### 2.1. Principle of Speckle Autocorrelation Imaging

In an incoherent imaging system, the object can be regarded as a luminous body composed of countless point light sources. After the spatially incoherent narrow-band light source illuminates the hidden object, the light that carries the object information is scattered through the scattering medium and received by the camera on the rear surface of the scattering medium, which ultimately results in the formation of a scattered image. Within the scope of the optical memory effect, the entire system has a point spread function that is invariant to spatial translation, and the image received by the whole camera can be understood as a superposition of the incoherent intensities of the scattering produced by each point light source. At this time, the speckle received by the camera can be expressed as

(1) I=O⊗S

where ⊗ denotes the convolution operation, O denotes the object function, and S denotes the PSF of the system. Calculating the autocorrelation of speckle, the following expression can be obtained:

(2) $$\begin{array}{ll} I★I & =\left(O⊗S\right)★\left(O⊗S\right)\\ & =\left(O★O\right)⊗\left(S★S\right) \end{array}$$

Since the autocorrelation of the scatter image point spread function $\left(S★S\right)$ is a peaked function, which is essentially the autocorrelation of broadband noise, we can obtain

(3) $$I★I=\left(O★O\right)+C$$

where C is the background constant term. According to the Wiener–Khinchin theorem, we can determine that

(4) $$I★I=FT^{-1}\left\{{\left|FT\left\{I\right\}\right|}^{2}\right\}$$

where FT and $FT^{-1}$ denote the Fourier transform and Fourier inverse transform. The power spectrum of the object can be obtained as follows:

(5) $${\left|FT\left\{O\right\}\right|}^{2}=FT\left\{O★O\right\}=FT\left\{I★I-C\right\}$$

Therefore, by calculating the autocorrelation of the speckle, we can obtain the Fourier amplitude of the hidden object. To recover the hidden object completely, we also need to calculate the Fourier phase of the hidden object in combination with the phase recovery algorithm. The most used traditional phase recovery algorithms are the Fienup-type phase-retrieval algorithm combined with the “Error reduction” (ER) algorithm and the “Hybrid Input–Output (HIO)” algorithm [34,35]. Since this algorithm is not the focus of this study, the algorithm flow will not be described in detail here.

**Figure 1:**
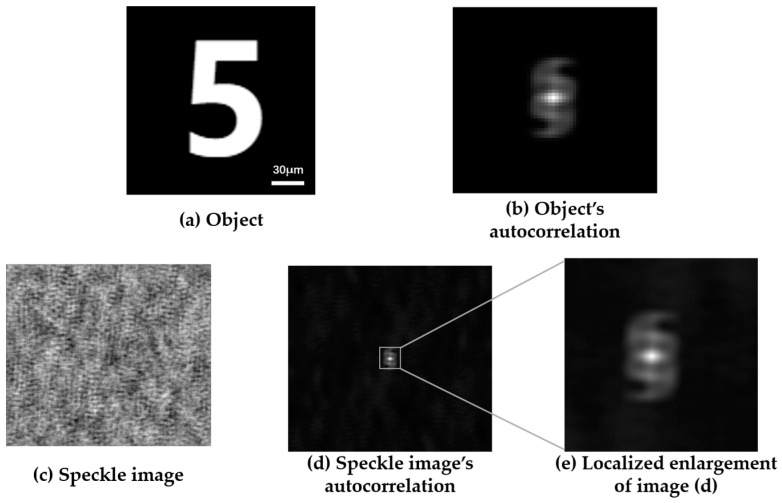
Speckle autocorrelation simulation results.

Simulations were performed based on the above speckle autocorrelation theory derivation and the results are shown in Figure 1. The light source wavelength is 632.8 nm, the scattering medium type is a random phase screen under ideal conditions, the detector pixel size is 2048 × 2048, the object size is 60 μm × 100 μm, and the speckle window size is 300 × 300. Figure 1a shows the original object image number “5”, and Figure 1c shows the speckle image received by the camera in the scattering imaging system. The autocorrelation of the original object was calculated directly, and the result is shown in Figure 1b. After the same calculation of the autocorrelation of the speckle image, the results were obtained as shown in Figure 1d. Figure 1e is the result after zooming in on the center area of Figure 1d. Comparing Figure 1b,e, we found that the autocorrelation results of the two were basically the same. Therefore, the conclusion that the Fourier amplitude information of the hidden object can be obtained by calculating the speckle autocorrelation was also verified.

**Figure 2:**
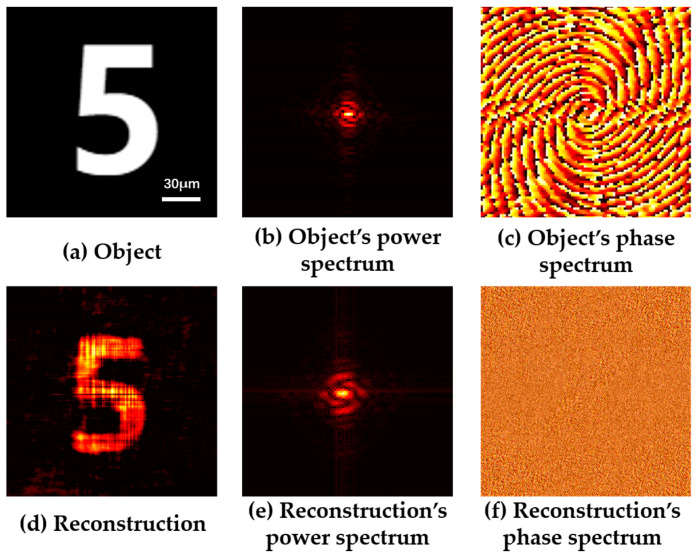
Numerical simulation results based on Fienup phase recovery scattering imaging method.

By inputting the obtained object’s Fourier amplitude information into the Fienup phase recovery algorithm, the object’s Fourier phase information can be obtained. Combining the Fourier amplitude information and Fourier phase information, the reconstructed object can be obtained. The numerical simulation results based on the Fienup phase recovery scattering imaging method are shown in Figure 2. Figure 2a shows the original hidden object, Figure 2d shows the reconstructed object calculated by the Fienup phase recovery algorithm, and Figure 2b compares the power spectrum of the original image with the amplitude information of the reconstructed object in Figure 2e, which can prove the accuracy of the restoration results.

It is worth mentioning that the window function was used to window the speckle autocorrelation results when computing the Fourier amplitude of the object. This is due to the limited range of the optical memory effect. The object was small compared to the size of the speckle image acquired directly by the camera, and when calculating the speckle autocorrelation, it was found that the object information only accounted for a very small part of the autocorrelation result. Therefore, when using autocorrelation information to recover the Fourier amplitude of an object, there is no need for an excessively high sampling frequency. On the contrary, an excessively high sampling frequency introduces high-frequency noise that affects the quality of the reconstruction. Therefore, the autocorrelation results were restricted using the window function, and only the central part was used to calculate the corresponding Fourier transform. In the subsequent content of this article, when other phase recovery algorithms are used to reconstruct hidden objects, this method is also used in the calculation of speckle autocorrelation.

### 2.2. Bispectrum Phase Recovery Algorithm

Traditional single-shot scattering imaging methods can image objects hidden behind the scattering medium, but the reconstruction results of the phase recovery algorithms used therein are strictly dependent on the accuracy of the object’s Fourier amplitude information. If the amplitude information is not calculated accurately, the hidden object cannot be reconstructed. The method utilizes a non-deterministic algorithm through repeated iterations which cannot obtain accurate object Fourier phase information, such as the actual direction of the object, and the imaging results are often not accurate enough. Therefore, a phase recovery method based on bispectrum analysis is proposed which can effectively avoid these problems.

The bispectrum analysis algorithm, which was widely used in atmospheric turbulence imaging in early years [36,37], is an indirect method for calculating the Fourier phase of objects. It is mainly divided into two steps: the calculation of the bispectrum and the phase reconstruction based on the bispectrum. The defining equation of the bispectrum is

(6) $$\begin{array}{ll} B\left(f_{1},f_{2}\right) & =\tilde{I}\left(f_{1}\right)\tilde{I}\left(f_{2}\right)\tilde{I}\left(-f_{1}-f_{2}\right)\\ & =\tilde{I}\left(f_{1}\right)\tilde{I}\left(f_{2}\right){\tilde{I}}^{*}\left(f_{1}+f_{2}\right) \end{array}$$

where $\tilde{I}\left(f\right)$ is the result of the Fourier transformation of the spatial domain speckle image $I\left(x\right)$ to the frequency domain, and $B\left(f\right)$ is the bispectrum value. In the atmospheric turbulence image restoration algorithm, the idea of averaging multiple shots in time is used to recover the object’s information. In this study, in the scattering imaging algorithm based on bispectrum analysis, the idea of averaging in space instead of averaging in time in the traditional algorithm was adopted. The object’s Fourier phase recovery was performed by cropping the scattering image directly acquired by the camera and then computing the bispectrum averaging method.

Each sub-speckle image obtained by cropping the camera speckle image can still be expressed as a convolution between the point spread function and the object according to the incoherent imaging formula, as with the direct collection of a speckle image.

(7) $${\langle B\left(f_{1},f_{2}\right)\rangle }_{N}=\tilde{O}\left(f_{1}\right)\tilde{O}\left(f_{2}\right){\tilde{O}}^{*}\left(f_{1}+f_{2}\right){\langle \tilde{S}\left(f_{1}\right)\tilde{S}\left(f_{2}\right){\tilde{S}}^{*}\left(f_{1}+f_{2}\right)\rangle }_{N}$$

where N is the number of sub-speckles after clipping, and ${\langle \tilde{S}\left(f_{1}\right)\tilde{S}\left(f_{2}\right){\tilde{S}}^{*}\left(f_{1}+f_{2}\right)\rangle }_{N}$ is the bispectrum transfer function. It can be seen from reference [5] that the bispectrum transfer function is a real function, and the influence of the bispectrum transfer function can be ignored when calculating the average bispectrum phase. The bispectrum transfer function does not include the aberration caused by the scattering medium and the optical system, so the object’s Fourier phase obtained by using bispectrum analysis is more accurate.

In addition to the computation of the bispectrum, another key step in the object’s Fourier phase recovery is phase reconstruction based on the bispectrum values. Since the bispectrum transfer function does not contain a phase term, by taking the phase on both sides of Equation (7), the phase relationship can be obtained as follows:

(8) $$\phi_{B}\left(f_{1},f_{2}\right)=\phi_{O}\left(f_{1}\right)+\phi_{O}\left(f_{2}\right)+\phi_{O}\left(-f_{1}-f_{2}\right)$$

where $\phi_{B}$ denotes the phase of the average bispectrum of the sub-speckle, and $\phi_{O}$ denotes the phase of the object. From Equation (8), it can be seen that the object phase can be derived sequentially from low to high frequencies by knowing $\phi_{O}$. However, some initial conditions need to be set; that is, the starting point of the recursion is determined, and the zero frequency is the starting point. According to the properties of the Fourier phase, the phase of the central frequency is 0; that is, $\phi_{O}\left(0\right)=0$. Meanwhile, since we only need to reconstruct the structural information of the object and not the positional information, we can set $\phi_{O}\left(1\right)=0$ to obtain the corresponding phase information of the remaining frequency positions of the object using the recursive method.

However, in the process of scattering imaging, we generally deal with 2D images, which will become 4D data when calculating the bispectrum:

(9) $$B\left(f_{1x},f_{1y},f_{2x},f_{2y}\right)=\tilde{I}\left(f_{1x},f_{1y}\right)\tilde{I}\left(f_{2x},f_{2y}\right){\tilde{I}}^{*}\left(f_{1x}+f_{2x},f_{1y}+f_{2y}\right)$$

where $\tilde{I}\left(f_{x},f_{y}\right)$ is the Fourier transform of the 2D speckle pattern. Taking an image with a size of 256 × 256 as an example, if it is calculated directly according to Equation (9), it will result in $256^{4}\approx 4\times 10^{9}$ elements. When performing bispectrum computation on high-resolution images, the huge amount of data will greatly affect the computing power. Therefore, without affecting the quality of the phase recovery, it is necessary to reduce the amount of bispectrum data calculations as much as possible and reduce the complexity of the entire imaging process.

Firstly, using the idea of Radon transform, the 2D speckle image is projected onto the 1D directions corresponding to different angles, and the original speckle image is transformed into multiple 1D scattering data for bispectrum computation as a way to improve the computational efficiency. The specific calculation of the Radon transform for the sub-speckle image is

(10) $$\tilde{I}\left(\rho ,\theta \right)=\int_{-\infty }^{\infty }I\left(\rho \cos\theta -s\sin\theta ,\rho \sin\theta +s\cos\theta \right)ds$$

where $\tilde{I}\left(\rho ,\theta \right)$ denotes the projection of the sub-speckle image $I\left(x,y\right)$ in the direction of a particular angle θ. At this time, the sub-speckle is transformed from the x−y coordinate to the ρ−s coordinate system, and all 2D sub-speckle data become 1D data. The algorithm in this study is specifically calculated by projecting each 2D sub-speckle image as 1D data on 18 angles distributed at equal intervals between 0 and 180°.

In order to further reduce the amount of data in bispectrum analysis, we used the method of discarding redundant data in bispectrum calculations and retaining only our region of interest. We call this process bispectrum truncation. Bispectrum truncation refers to a loss of image information. Methods of retaining valid data and deleting redundant data need to be considered from the following perspectives: (1) bispectrum areas close to the neighborhood of the coordinate axes have the highest signal-to-noise ratio; (2) in order to overcome the effects of anisoplanatism, a larger combination of $f_{1}$, $f_{2}$, and $f_{1}+f_{2}$ should be selected; and (3) in order to make the subsequent calculations simple, the selected region of the bispectrum should not be too dispersed and should consist of a continuous region.

**Figure 3:**
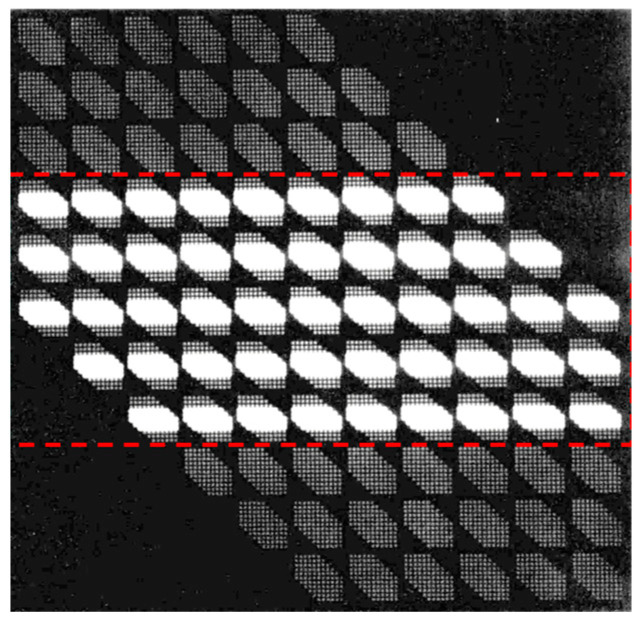
Schematic diagram of bispectrum truncation.

In this study, the truncation method proposed by Pehlemann et al. was used [38]. When the 1D data that need to be calculated as a bispectrum value are $\tilde{I}\left(f\right)$, the range of f is limited to

(11) $$\left|f\right|\leq 0,\ldots ,\left(1-C\right)\times M$$

where C is a parameter that regulates the range of truncated data, and M is the size of the data after the Randon transform of the sub-speckle image. The value of C should range from 0 to 1. When C takes a larger value, more bispectrum data are truncated, and when C takes a smaller value, less bispectrum data are truncated. Figure 3 shows a schematic of the effect of bispectrum truncation when *M* = 10. As shown in the figure, the original bispectrum data is four-dimensional data. Two of the four bispectrum coordinates are chosen to form a two-dimensional outer matrix, which represents a two-dimensional sub-plane of the spectrum. Then the remaining two bispectrum coordinates are generated into an inner matrix and used as the filling elements of the outer matrix to obtain a four-dimensional bispectrum data matrix, which is the dark particle part in the figure. After processing the data using the bispectrum truncation method, the redundant data were removed based on the truncation parameters, and the bispectrum data were retained for continued computation. It can be seen that the data from both the outer and inner matrices are processed at the same time, that is, the part of the bright particles within the red frame in the figure. The effect of the choice of specific truncation parameters on object phase recovery and reconstructed image quality is discussed in detail in Section 4.1.

**Figure 4:**
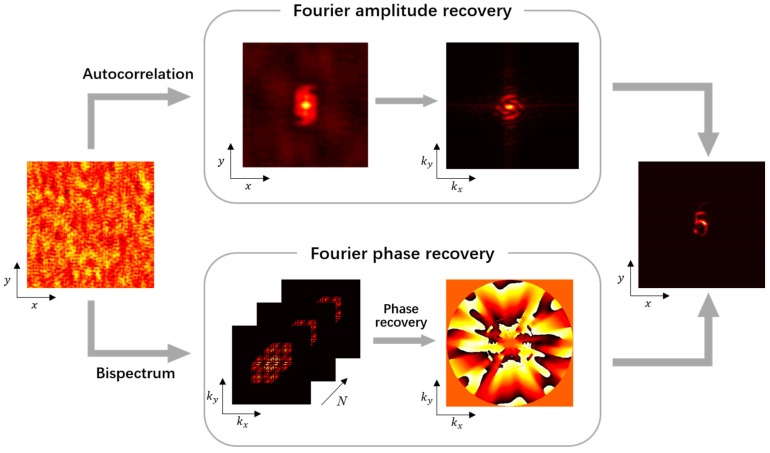
Flowchart of single-shot speckle imaging algorithm based on bispectrum truncation.

Based on the above theoretical analysis, the overall process of the single-shot speckle imaging algorithm based on bispectrum truncation that we propose is shown in Figure 4. The Fourier amplitude of the object was obtained by calculating the speckle autocorrelation, and the object’s Fourier phase was extracted by calculating the bispectrum averaging and phase reconstruction from the cropped sub-speckles. The method does not require a large number of repeated iterative calculations, the processes of acquiring the amplitude and phase information are independent of each other, and the accuracy of phase recovery does not depend on the accuracy of its amplitude estimation. Additionally, the amount of data and complexity that need to be computed are greatly reduced by Randon transform and bispectrum truncation, and the reconstruction of the hidden object can be achieved by obtaining the Fourier amplitude and phase of the object from the speckle image collected directly by the camera.

## 3. Experiment Platform

**Figure 5:**
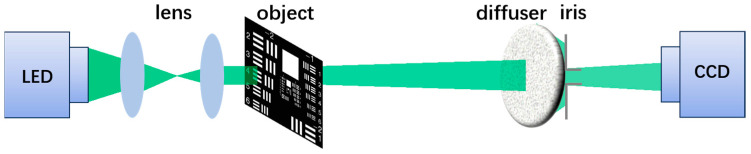
Schematic diagram of experimental light path.

**Figure 6:**
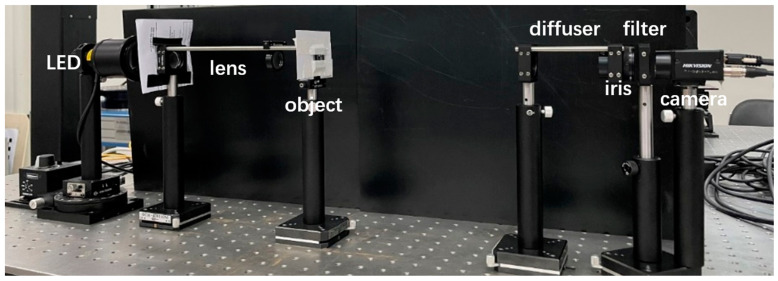
Physical diagram of the experimental light path.

In our experiments, the spatially incoherent light source was composed of a LED light source (Thorlabs, Newton, NJ, USA, M530L4, center wavelength 530 nm, bandwidth 35 nm) and two collimating lenses, and the imaging object was a negative resolution test plate. The incoherent light illuminated the object, the transmitted light carried the object information to the diffuser (Thorlabs, DG10-600), and the object was about 64 cm away from the diffuser. An iris with a diameter of about 2.5 mm was placed on the other side close to the diffuser to control the image contrast of the received camera speckle. After the transmitted light was scattered by the diffuser, the image was directly collected by a high-resolution camera (HIKVISION, Tokyo, Japan, MV-CB060, pixel size 2.4 um, resolution 3072 × 2048). The camera was placed about 8.5 cm behind the diffuser. In order to ensure the contrast of the speckle image collected directly by the camera, we added a narrow-band filter (Thorlabs, FLH532-4) with a bandwidth of about 4 nm in front of the camera. The schematic diagram of the light path structure of the optical system for incoherent imaging through thin scattering media is shown in Figure 5, and the actual picture of the experimental light path is shown in Figure 6.

**Figure 7:**
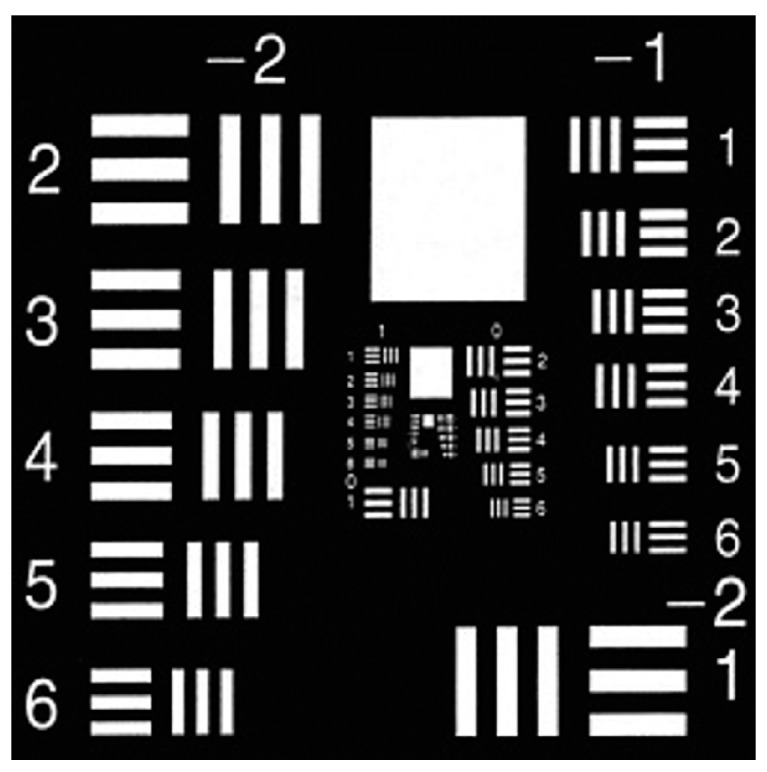
Picture of the resolution test board.

**Figure 8:**
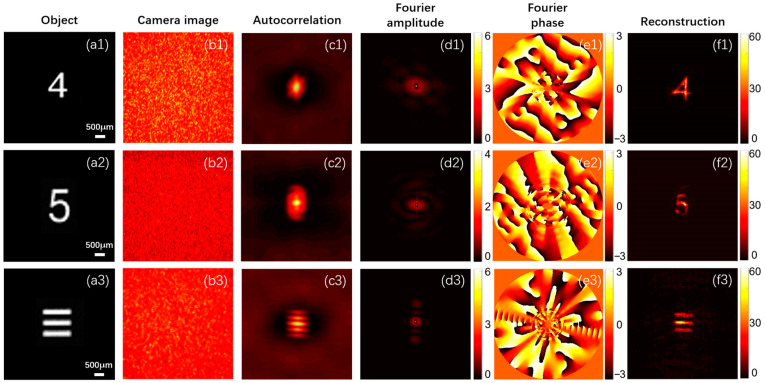
Experimental results based on the bispectrum truncation method. (**a1**–**a3**): Original object images. (**b1**–**b3**): Original speckle images of different objects. (**c1**–**c3**): Autocorrelation of speckle images. (**d1**–**d3**): Fourier amplitudes of different objects. (**e1**–**e3**): Fourier phases obtained by the bispectrum truncation method. (**f1**–**f3**): Reconstructed images (the result of the truncation parameter *C* = 0.7).

The resolution test plate is shown in Figure 7. The objects used in this experiment were ‘4’, ‘5’, and ‘≡’ in Group 1 of the resolution test plate, as shown in Figure 8(a1–a3).

Due to the limitation of the optical memory effect, the field of view (FOV) of the whole imaging system is limited by the range of the optical memory effect, so the maximum range of the field of view of the imaging system is

$$\Delta x=\frac{u\lambda }{\pi l}$$

where u denotes the distance between the object and the diffuser, λ denotes the wavelength of the illuminating light, and l denotes the thickness of the scattering medium.

The resolution of an imaging system is primarily derived from the diffraction limit of the system. For random scattering imaging systems, the effective incident pupil is adjusted by setting an iris behind the scattering medium. The minimum resolvable interval on the image plane is

$$\delta =1.22\frac{\lambda d}{D}$$

where λ denotes the wavelength of the illumination light, d denotes the distance from the aperture diaphragm to the imaging plane, and D denotes the diameter of the aperture diaphragm. Therefore, when the detector pixel size is larger than δ, it will cause reconstruction distortion or even failure to reconstruct. After calculations, the sizes of the objects were within the range of the optical memory effect zone.

Figure 8(b1–b3) show the original speckle images collected by the camera when imaging different objects. It can be seen that they are typical messy speckle patterns. The original speckle image was preprocessed and filtered, and then the autocorrelation was calculated. Figure 8(c1–c3) show the results after restricting the original speckle autocorrelation using the window function. In order to fully suppress the statistical noise of the bispectrum and extract the object’s Fourier phase from the single-shot speckle image, we cropped the single-shot speckle image to obtain multiple sub-speckle images and filtered each sub-speckle image using a Gaussian windowing function. Then, we analyzed the bispectrum using the truncation method described in the previous section to obtain the Fourier phase of the object. Figure 8(d1–d3) show the Fourier amplitudes of the reconstructed objects. Figure 8(e1–e3) show the recovered Fourier phases when each sub-speckle image was 300 × 300 in size. Therefore, the Fourier distribution of each object image can be obtained from the Fourier amplitude and phase, and the recovered object can be obtained by calculating the Fourier inverse transform. Figure 8(f1)–(f3) show the object reconstruction results based on the bispectrum truncation method.

## 4. Discussion of Experimental Results

### 4.1. Effect of Different Truncation Parameters on Object Reconstruction

**Figure 9:**
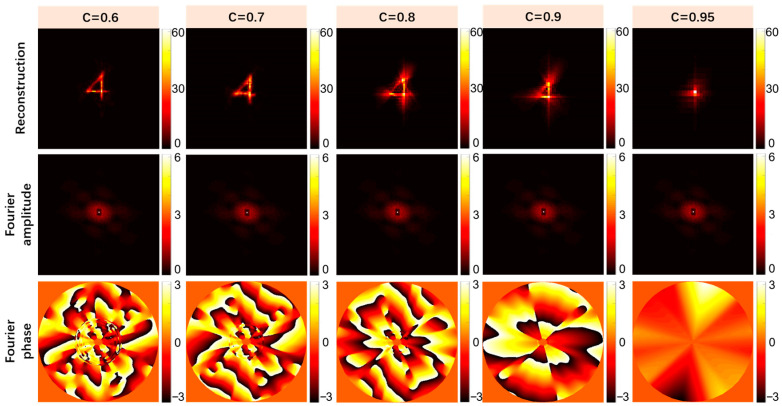
Schematic diagram of object reconstruction effect under different truncation parameters.

**Figure 10:**
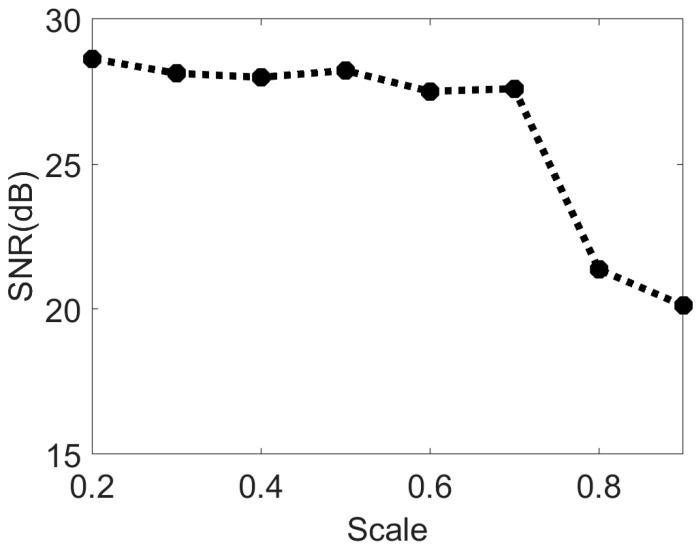
Schematic diagram of the relationship between the SNR and the variation in the truncation parameter.

In order to quantitatively analyze the impact of truncation parameters on the object reconstruction effect, we compared the reconstruction results of speckle images under different truncation parameters with the original objects and introduced the SNR (signal-to-noise ratio) to evaluate the object reconstruction quality under different truncation parameters. The larger the truncation parameter, the fewer bispectrum data were retained. As shown in Figure 9, when the truncation parameter continued to increase, the intercepted data also continued to increase and the reconstruction quality of the object decreased. Figure 10 shows the relationship between the evaluation index SNR and the truncation parameter. When the truncation parameter was less than 0.7, the SNR did not change significantly with the truncation parameter. After the truncation parameter C was increased to 0.7, the quality of the object reconstruction was significantly reduced, which indicates that in addition to the redundant data, there were key data in the bispectrum phase that were intercepted, which led to the object not being reconstructed. Combined with the SNR of the reconstructed image and the amount of comprehensively computed data, when the truncation parameter *C* = 0.7, the computation amount could be minimized without damaging the reconstruction quality of the object, and the optimal solution was achieved between the reconstruction effect and the cost of data computation.

As shown in Table 1, when the truncation parameter *C* = 0.7, compared with using the original bispectrum data for phase analysis, the algorithm in this paper reduces the calculation amount of bispectrum data by about 80%. And the overall algorithm time for object reconstruction was reduced by about 30%. In the following experiments, *C* = 0.7 was used for bispectrum data interception if not specifically indicated.

### 4.2. Effects of Media with Different Scattering Degrees on the Quality of Object Reconstruction

**Figure 11:**
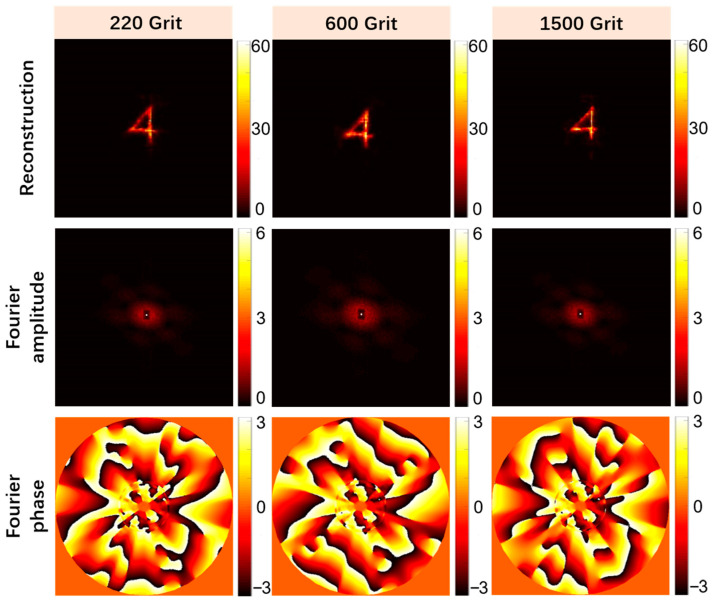
Comparison of the recovery effects of speckle images collected by media with different scattering degrees.

In order to evaluate the reconstruction ability of our algorithm for hidden objects under scattering media possessing different scattering degrees, three different mesh sizes of woolen glass were used as diffusers for our comparative experiments. The models used were Thorlabs, DG10-220, DG10-600, and DG10-1500. The other settings in the experimental optical path were the same as those described in Section 3. Only the diffuser was changed, the object reconstruction of the collected speckle images was performed, and the reconstruction effect was evaluated. Figure 11 illustrates the object reconstruction results of the speckle images acquired using three different diffusers. It can be seen that the hidden objects were reconstructed well at either larger (DG10-1500) or smaller (DG10-220) mesh sizes. Table 2 shows the SNR of the reconstructed objects for the three cases, and the SNR remained above 25 dB under different scattering degree conditions. This shows that this algorithm possesses a good ability to reconstruct hidden objects for media with different scattering degrees under the current optical path structure configuration.

The scattering medium used in this experiment is ground glass with different grits. It can also be replaced with other types of scattering media, such as sliced chicken breasts, the inner skin of onions, etc., which have been verified in other references.

### 4.3. Statistical Noise Analysis in Bispectrum Reconstruction Methods

The basic idea of the bispectrum analysis used in the phase recovery process of this study is based on the theory of phase closure in atmospheric imaging. Traditional bispectrum analysis uses multi-shot speckles for statistical averaging, and both the signal amplitude and the bispectrum statistical noise amplitude are elevated with an increase in the number of frames. During the imaging process, a sufficiently high bispectrum SNR needs to be ensured in order to obtain satisfactory imaging quality. The SNR of this study’s algorithm for phase recovery to compute the bispectrum can be expressed as $N^{1/2}/n^{1/2}$, while the SNR for amplitude recovery to compute the autocorrelation is $N^{1/2}$, where N is the number of frames [39]. Since this study adopted the method of single-shot speckle image recovery, here N can be understood as the number of speckle particles, and n is the number of speckle particles contained in each sub-speckle frame. In other words, the SNR increases with the number of speckle particles. We found that when other optical path conditions were the same, the SNR of the bispectrum signal was lower than the SNR of the autocorrelation signal.

**Figure 12:**
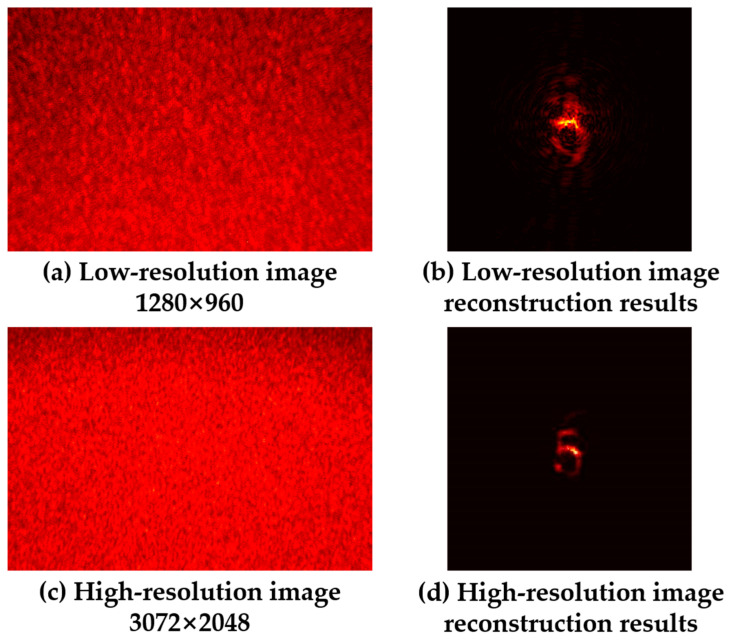
Comparison of reconstruction results of speckle images with different resolutions.

Figure 12 shows the use of two cameras with different resolutions (HIKVISION, MV-CE013, resolution 1280 × 960; HIKVISION, MV-CB060, resolution 3072 × 2048) to collect speckle images when other light path conditions remained the same, using the object reconstruction results based on the bispectrum truncation method to obtain phase information. It can be seen that the low-resolution speckle image was unable to reconstruct the hidden object. Therefore, when we need to reconstruct an object of the same size, the phase recovery method using bispectrum analysis requires more speckle particles to participate in the statistical average; that is, a higher-resolution speckle image is required.

### 4.4. Lens-like Properties of Scattering Media

**Figure 13:**
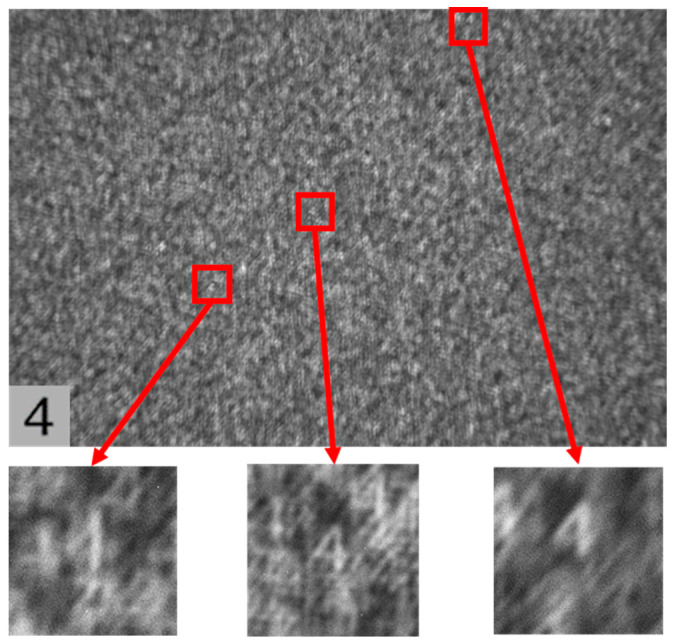
Direct observation of the self-imaging of the object ‘4’ in the original speckle image.

**Figure 14:**
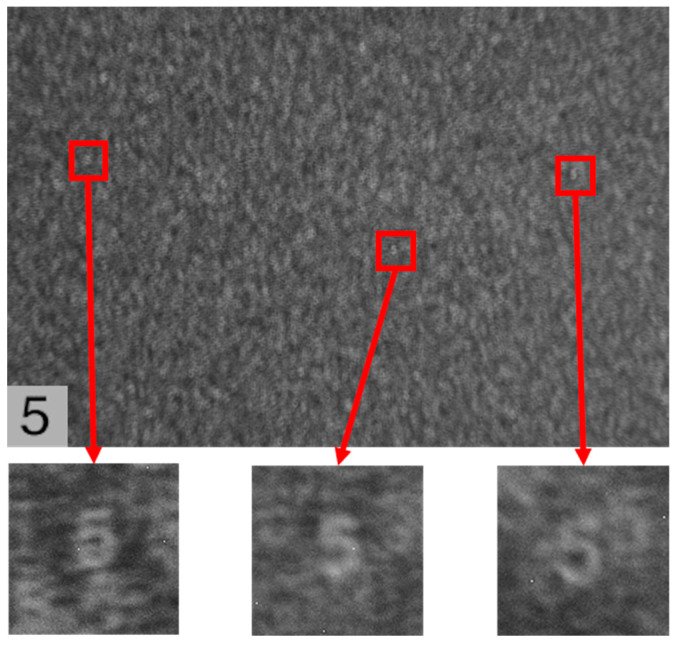
Direct observation of the self-imaging of the object ‘5’ in the original speckle image.

In the experiments described in the previous section, typical grainy and scrambled patterns were observed in the speckle images acquired directly from the camera, and complex computations, such as cropping, filtering, and phase restoration, were performed on the original speckle images to reconstruct the hidden objects. Interestingly, however, by zooming in on the original speckle images captured in the experiment, we found that this extra computational work was not strictly necessary and that hidden object images with clear contours could be obtained through direct observation. As shown in Figure 13 and Figure 14, the small image below is a zoomed-in view of the red region of the original speckle image, from which the self-imaging of the original hidden objects ‘4’ and ‘5’ can be recognized.

In fact, in the optical system of scattering imaging, the object information is not lost but is encoded after the incident light has been scattered several times by the diffuser. Therefore, according to the relationship between the hidden object and the speckle, the diffuser can be treated as a collection of distributed lenses with a random phase mask. The speckle can be similarly treated as the self-imaging of a hidden object through a collection of distributed lenses with a random phase mask. The image made by each cell is overlapped and combined, eventually forming a scrambled speckle. Therefore, we can obtain a partially complete image modulated by the phase of the diffuser using the scattering map acquired directly by the camera. This makes it possible to observe hidden objects using direct imaging through the diffuser.

## 5. Conclusions

In this study, a single-shot scattering medium imaging method based on the truncation method is proposed. First, the theoretical derivation and data simulation of the method are carried out, and the overall computational flow of the algorithm is given. The method allows the Fourier phase of the hidden object to be obtained using bispectrum analysis. Compared with the traditional method, it avoids a large number of repeated iterative calculations and the uncertainty of the reconstruction results, and intercepts the redundant data when calculating the bispectrum data, which greatly reduces the amount of data computation required. Secondly, a scattering imaging optical experimental platform was built to experimentally validate the method. Using the signal-to-noise ratio (SNR) as the target reconstruction quality evaluation index, the imaging capability of this method was quantitatively evaluated. The experimental results show that our method can achieve both computational minimization without damaging the quality of the reconstructed object and an optimal solution between the reconstruction effect and the cost of data computation. Compared with the use of original bispectrum data for phase analysis, the amount of bispectrum data to be calculated was reduced by about 80%, and the overall algorithm time for object reconstruction was reduced by about 30%. Finally, we also quantitatively assessed the effect of different truncation parameters and different degrees of diffuser on the quality of object reconstruction. The statistical noise and scattering medium properties in the experiment were also analyzed, which may provide new ideas for other novel imaging methods.

## Figures and Tables

**Table 1:** Results of quantitative comparison between this paper’s algorithm and traditional algorithms.

	Data Calculation Amount	Time (s)	SNR (dB)
Traditional method	100%	222.21	27.99
Our method	20.25%	152.88	27.60

**Table 2:** SNR of reconstructed objects under different scattering levels.

Diffuser	SNR (dB)
220 Grit	26.95
600 Grit	27.60
1500 Grit	29.24

## Data Availability

Data are contained within the article.
